# Genetic and biological characteristics of species A rotaviruses detected in common shrews suggest a distinct evolutionary trajectory

**DOI:** 10.1093/ve/veac004

**Published:** 2022-01-28

**Authors:** Alexander Falkenhagen, Simon H Tausch, Anton Labutin, Josephine Grützke, Gerald Heckel, Rainer G Ulrich, Reimar Johne

**Affiliations:** Department of Biological Safety, German Federal Institute for Risk Assessment, Max-Dohrn-Str. 8-10, Berlin 10589, Germany; Department of Biological Safety, German Federal Institute for Risk Assessment, Max-Dohrn-Str. 8-10, Berlin 10589, Germany; Institute of Ecology and Evolution, University of Bern, Baltzerstrasse 6, Bern CH-3012, Switzerland; Department of Biological Safety, German Federal Institute for Risk Assessment, Max-Dohrn-Str. 8-10, Berlin 10589, Germany; Institute of Ecology and Evolution, University of Bern, Baltzerstrasse 6, Bern CH-3012, Switzerland; Institute of Novel and Emerging Infectious Diseases, Friedrich-Loeffler-Institut, Federal Research Institute for Animal Health, Südufer 10, Greifswald-Insel Riems 17493, Germany; Deutsches Zentrum für Infektionsforschung (DZIF), Partner site Hamburg-Lübeck-Borstel-Riems, Südufer 10, Greifswald-Insel Riems 17493, Germany; Department of Biological Safety, German Federal Institute for Risk Assessment, Max-Dohrn-Str. 8-10, Berlin 10589, Germany

**Keywords:** rotavirus, shrew, phylogeny, evolution, reassortment, zoonosis, reverse genetics

## Abstract

Species A rotaviruses (RVAs) are important aetiological agents of severe diarrhoea in young children. They are also widely distributed in mammals and birds, and increasing evidence indicates the possibility of zoonotic transmission of RVA strains between animals and humans. Moreover, reassortment of the eleven segments of the RVA genome can result in rapid biological changes and may influence pathogenic properties. Here, the nearly complete genome of an RVA strain from a common shrew (*Sorex araneus*) was sequenced, which showed high nucleotide sequence similarity to additionally determined partial sequences from common shrew RVAs but only very low identity (below 68 per cent) to RVAs from other animal species and humans. New genotypes were assigned to most genome segments of the novel common shrew RVA strain KS14/269, resulting in the genome constellation G39-P[55]-I27-R26-C22-M22-A37-N26-T26-E30-H26. Phylogenetic analyses clustered the common shrew RVAs as ancestral branches of other mammalian and avian RVAs for most of the genome segments, which is in contrast to the phylogeny of the hosts. Nevertheless, conserved sequences typical for all RVAs were identified at the 5ʹ- and 3ʹ- non-coding segment termini. To explore whether the common shrew RVA can exchange genetic material with other mammalian RVAs by reassortment, a reverse genetics system based on the simian RVA strain SA11 was used. However, no viable reassortants could be rescued by exchanging the VP4-, VP6-, or VP7-encoding genome segment alone or in combinations. It can be concluded that highly divergent RVAs are present in common shrews, indicating an evolution of these viruses largely separated from other mammalian and avian RVAs. The zoonotic potential of the virus seems to be low but needs to be further analysed in future.

## 1. Introduction

Rotaviruses are enteric viruses that can cause severe gastroenteritis in young children. It has been estimated that 128,500 deaths were attributed worldwide to rotavirus infection among children younger than 5 years in 2016 (Troeger et al. 2018). In addition, rotaviruses are also widely distributed in domestic (Otto et al. 2012, 2015) and some wild animal species (Sachsenröder et al. 2014; Moutelíková et al. 2016).

Rotaviruses are composed of a non-enveloped triple-layered virus capsid, which encloses a genome of eleven segments of double-stranded RNA and the viral RNA replication enzymes VP1 and VP3. The genome segments can be exchanged by reassortment during simultaneous infection of a cell by two rotaviruses of the same species, which increases genetic variability (McDonald et al. 2016). Each genome segment encodes one or two viral proteins, resulting in six viral structural proteins (VP1–VP4 and VP6–VP7) and five or six non-structural proteins (NSP1–NSP6). VP2 forms the inner shell and VP6 the middle layer of the virus capsid. VP4 and VP7 are situated at the outer layer of the capsid and contain the major antigenic epitopes capable of eliciting neutralizing antibodies (Desselberger 2014). VP4, VP7, and VP6 interact with each other in the mature rotavirus particle (Settembre et al. 2011).

All rotaviruses are classified into the family *Reoviridae*, genus *Rotavirus* (International Committee on Taxonomy of Viruses 2020). Based on VP6 amino acid sequence identity, rotavirus species A–J have been defined (Matthijnssens et al. 2012) and the putative additional species K and L have recently been described (Johne et al. 2019). Among them, rotavirus A (RVA) has been given the highest attention as it is responsible for most cases of gastroenteritis in humans and animals. Within RVA, genome segments are assigned to genotypes based on specific nucleotide sequence identity cut-offs (Matthijnssens et al. 2008a,b), which yielded so far 41 G types (for the glycosylated VP7) and 57 P types (for the protease-sensitive VP4) (Rotavirus Classification Working Group 2021). The other genome segments have similar numbers of genotypes reflecting the high genetic diversity of RVA strains.

There is increasing evidence of zoonotic transmission of rotaviruses between different animal species and humans, which contributes to the high genetic diversity of rotaviruses in humans (Martella et al. 2010; Simsek et al. 2021). In some cases, interspecies transmission of RVAs is considered to result in the establishment of new virus lineages within the new host. For example, G9 and G12 RVA strains emerged in the human population during the last two decades, and phylodynamic sequence analyses suggested transmission events between pigs and humans in 1989 and 1995, respectively (Matthijnssens et al. 2010). Besides transmitted animal strains, reassortants containing a mixture of human and animal RVA genome segments have been identified (Martella et al. 2010; Tamim et al. 2019). However, only a small proportion of theoretically possible reassortants between RVA strains have been observed so far (Graham et al. 1987). Incompatibilities between the genome segments or their products might explain this observation, leading to reduced fitness or complete loss of viability (Falkenhagen et al. 2019; Patzina-Mehling et al. 2020). Recently, a reverse genetics system has been established for the simian RVA strain SA11, which allows a more detailed analysis of reassortment requirements through site-directed mutagenesis and targeted exchanges of genome segments (Kanai et al. 2017; Komoto et al. 2018; Falkenhagen et al. 2020).

The long-term evolutionary history of RVA is still poorly understood. Phylogenetic analyses indicated a general grouping of avian and mammalian RVA strains into different clusters (Trojnar, Otto, and Johne 2009). However, strains from a fox and a raccoon clustering with avian strains have been recently described, which might be a result of sporadic virus transmission by contact or ingestion of birds (Busi et al. 2017). Besides the mammalian/avian clustering, no further large phylogenetic groupings are evident within RVA. Many RVA genotypes are typically associated with specific animal species, which may indicate host-specific strain adaptation (Sen et al. 2009; Feng et al. 2013), but zoonotic transmissions to other hosts also exist. It has therefore been proposed that the evolution of human and animal rotaviruses is intersected by multiple, repeated events of interspecies transmission and subsequent strain adaptation (Martella et al. 2010). Recently, RVA strains have been identified in bats, which show a large diversity within the mammalian cluster, pointing to a possible origin of several mammalian RVA strains in bats (Simsek et al. 2021). In addition, reassortment events can influence the evolution of RVAs. For instance, avian RVA strains have a NSP1 sequence closely related to that of rotavirus species D, indicating that, sometimes in the past, the avian RVAs received their NSP1-encoding genome segment from a virus related to species D (Trojnar et al. 2010).

Shrews are small insectivore mammals that belong to the family Soricidae, order Eulipotyphla (Wilson and Mittermeier 2018). RVA strains have been first detected in Asian house shrews (*Suncus murinus*) in China, which were characterized by a relatively close relationship to other mammalian RVAs (Li et al. 2016). In contrast, an initial characterization of RVAs from common shrews (*Sorex araneus*) in Germany in 2019 indicated an only distant relationship of these RVAs to that of other mammals (including Asian house shrew) and birds; however, the complete genomes were not determined at this time point (Johne et al. 2019).

The aim of this study was to assess the distinct relationship of the RVA strains from common shrews with that of other hosts and to estimate their potential to create viable reassortants with other RVAs. To this end, one nearly complete genome sequence and additional partial genome sequences of common shrew RVA strains were determined and compared to other RVA sequences. Phylogenetic analyses were applied to elucidate the relationship and evolution of the common shrew RVAs in the context of other RVA strains and their hosts. In addition, biological data on the reassortment potential of the common shrew RVA with a simian RVA strain were generated using a reverse genetics system. The results support the common shrew RVAs as a remarkably divergent evolutionary lineage compared to other RVAs, with an estimated low zoonotic potential.

## 2. Materials and methods

### 2.1 Shrew samples and RNA isolation

A total of sixteen shrews were trapped between 2012 and 2013 in the Baden-Wuerttemberg region of Germany (Obiegala et al. 2017). Details on the samples are provided in Supplementary Data S1. Intestines were eviscerated and stored at −20°C until analysis. For the isolation of RNA, samples were thawed, and 1 ml phosphate buffered saline (PBS)/g intestine was added and vortexed for 30 s. After centrifugation at 5,000 × *g* for 10 min, RNA was isolated from 140 µl of the resulting faecal suspension using the QIAamp Viral RNA Mini Kit (Qiagen, Hilden, Germany). RNA was stored at −80°C until further analysis.

### 2.2 RT-PCR detection of shrew RVA

Our first approach to RVA detection used two established real-time RT-PCR protocols, allowing the detection of a wide range of mammalian RVAs (Pang et al. 2004; Otto et al. 2015). In a second approach, primers were delineated from a set of available mammalian and avian RVA sequences for the VP1-encoding genome segment, along with a previously determined corresponding sequence of an RVA from a common shrew (GenBank no. MN307986). The resulting primers generating a 387 base-pair (bp) fragment are shown in Supplementary Data S2. Conventional RT-PCR was performed using the QIAGEN One Step RT-PCR Kit (Qiagen). The temperature profile included reverse transcription at 42°C for 30 min, enzyme activation at 95°C for 15 min, followed by forty cycles with denaturation at 94°C for 30 s, annealing at 56°C for 30 s, and elongation at 74°C for 40 s, followed by a final elongation at 74°C for 5 min. PCR products were analysed on ethidium bromide-stained agarose gels.

### 2.3 Whole-genome sequencing of shrew RVA strains

A total of 20 μl of the extracted RNA of samples KS13/718 and KS14/269 was each mixed with 5 μl nuclease-free water, 3 μl 10× DNase buffer, and 2 μl TurboDNase (Invitrogen). After incubation at 37°C for 30 min followed by enzyme inactivation at 65°C for 10 min, RNA was purified using MobiSpin S-400 columns (MoBiTec, Göttingen, Germany). The resulting RNA preparation was thereafter subjected to reverse transcription and random amplification using the WTA2 Whole Transcriptome Amplification Kit (Sigma, Deisenhofen, Germany) as described (Johne et al. 2019). The DNA was fragmented to an average size of 400 bp with the M220 focused-ultrasonicator (Covaris, Brighton, UK) and subjected to library preparation using the TruSeq Nano DNA Library Prep kit (Illumina) according to the manufacturer’s instructions. Resulting DNA libraries were sequenced with 2 × 150 cycles with the NextSeq 500 (Illumina) and for each sample ∼1 million reads were obtained.

Raw reads underwent quality control and trimming using the AQUAMIS pipeline v.1.2.0 (Deneke et al. 2021) in mode—no_assembly. Trimmed reads were assembled using SKESA v.2.4.0 (Souvorov, Agarwala, and Lipman 2018), resulting in 32 and 146 contigs for samples KS14/269 and KS13/679, respectively. The contigs were searched in database Refseq (Brister et al. 2015) containing all rotavirus sequences available at NCBI (downloaded on 27 September 2021) using BLAST v.2.5.0. (Camacho et al. 2008). The eleven contigs with the highest bitscores were selected as Rotavirus segments and subjected to further analyses. The termini of the contigs from sample KS14/269 were elongated by iteratively mapping the trimmed reads to the contigs and manually selecting additional ∼30–100 bp per contig where no significant ambiguities appeared using Geneious Prime® 2020.2.2 (https://www.geneious.com).

In order to complete the sequences of the open reading frames and to confirm sequences at selected genome segment termini for sample KS14/269, conventional RT-PCRs were performed combined with Sanger sequencing of the PCR products. Primer sequences were determined from alignments of selected mammalian and avian RVA sequences and from the next-generation sequencing (NGS) derived sequence of strain KS14/269, which are shown in Supplementary Data S2. RT-PCRs were performed using the QIAGEN One Step RT-PCR Kit (Qiagen). For the amplification of the 5ʹ-end of the VP4-encoding genome segment, a rapid amplification of cDNA ends (RACE) approach was used by the application of the 5ʹ-RACE System (Invitrogen) with primers S4-200-as and S4-173-as (Supplementary Data S2). The products of RT-PCR and RACE were directly Sanger sequenced by a commercial company (Eurofins Genomics Germany GmbH, Ebersberg, Germany) using the amplification primers. The final genome sequence of RVA strain KS14/269 was assembled from the NGS contigs and the Sanger sequences of the amplified fragments using the SeqBuilder module of the DNASTAR software package (Lasergene, Madison, WI, USA). The sequences were submitted to the GenBank database with accession numbers MZ054764–MZ054774 for the strain KS14/269 genome and MZ099618–MZ099628 for the genome segment fragments of strain KS13/718.

### 2.4 Sequence comparison

Genotyping of the genome segments was first attempted using the Rotavirus A Genotype Determination Tool ViPR pathogen database (https://www.viprbrc.org/brc/rvaGenotyper.spg?method=ShowCleanInputPage&decorator=reo), which resulted in the suggestion to contact the Rotavirus Classification Working Group (RCWG) regarding the assignment of novel genotypes. Therefore, all segment sequences were submitted to RCWG and novel genotypes were assigned. For further sequence comparisons, all RVA genotype reference sequences were used, which were publicly available at GenBank database according to the list of accepted genotypes available at the RCWG website (https://rega.kuleuven.be/cev/viralmetagenomics/virus-classification/rcwg, accessed 29 March 2021). In addition, the reference sequences for rotavirus species D, F, and G were included as outgroups. For the determination of sequence distances and identities, the Clustal W algorithm implemented in the MegAlign Pro module of the DNASTAR software package (Lasergene) was used.

### 2.5 Phylogenetic analysis

Rotavirus nucleotide sequences were highly variable and differed extensively in length and thus the initial alignment was based on their inferred amino acid sequences (coding regions only) using ClustalW (Thompson, Higgins, and Gibson 1994) (gap open: 10.00, gap extend: 0.2). This approach enables typically more reliable identification of the positions of gaps in highly variable nucleotide sequence alignments (Kindler et al. 2013). Phylogenetic reconstructions then used back-translated nucleotide sequence alignments obtained with PAL2NAL program (Suyama, Torrents, and Bork 2006), which preserved the original nucleotides also at synonymous positions. A phylogenetic analysis was performed with MrBayes v3.2.7 (Ronquist et al. 2012) on the CIPRES platform (Miller, Pfeiffer, and Schwartz 2010). Metropolis-coupled Markov chain Monte Carlo sampling was performed for 10^8^ generations in four independent runs comprising four chains with seed set to 111. We implemented reversible-jump sampling over the entire general time-reversible substitution model space (Huelsenbeck, Larget, and Alfaro 2004), and samples were recorded every 10^3^ generations after discarding a burn-in fraction of 25 per cent. Phylogenetic trees were drawn and edited using the online platform iTOL v5 (Letunic and Bork 2019). Phylogenetic relationships between host species were estimated using the TimeTree online resource (Kumar et al. 2017). TimeTree utilizes a consensus tree of life with over 97,000 species based on over 3,000 manually curated phylogenetic and molecular evolution studies, which report estimates of divergence times among species (Hedges et al. 2015; Kumar et al. 2017). Polytomies within the timetree were resolved by mapping it against a conservative guidetree of the National Center for Biotechnology Information community consensus taxonomy (Hedges et al. 2015; Sayers et al. 2021).

### 2.6 Construction of plasmids

The plasmids encoding the eleven SA11-L2 genome segments as well as the three helper plasmids pCAG-D1R, pCAG-D12L, and pCAG-FAST-p10 (Kanai et al. 2017) were obtained from Addgene (Watertown, MA, USA). The shrew RVA strain KS14/269 VP4-, VP7-, and VP6-encoding plasmids (GenBank: MZ054767, MZ054772, and MZ054769, respectively) contained an expression cassette consisting of a T7 RNA polymerase promoter, the complete genome segment, a hepatitis delta virusoid ribozyme sequence, and a T7 RNA polymerase terminator at the 3ʹ-end. The sequences for the promoter, ribozyme, and terminator are identical to that of a plasmid encoding VP4 from avian RVA strain 02000V2G3 (GenBank: KT239165, Johne et al. 2015) and a plasmid encoding VP4 from bat RVA strain BatLy03 (Falkenhagen et al. 2019). The expression cassettes were synthesized as gBlocks gene fragments by Integrated DNA Technologies (IDT, Coralville, IA, USA). Adenosine overhangs were added to the gBlocks using Takara Ex Taq (Takara Bio Inc, Kusatsu, Japan) and the fragments were cloned into pCR4-TOPO using a TOPO TA cloning kit (Thermo Fisher Scientific, Waltham, CA, USA) according to the manufacturer’s instructions. The sequence of the expression cassette was verified by Sanger sequencing (Eurofins Genomics Germany GmbH). The expression cassettes were then cloned into pUCIDT-Amp (IDT) using traditional cloning methods. All plasmids were purified using a plasmid midi kit (Qiagen, Hilden, Germany) prior to transfection.

### 2.7 Attempts to generate reassortants using reverse genetics

Attempts to generate reassortants using a reverse genetics system were essentially performed as described previously (Falkenhagen et al. 2020). Briefly, 80–90 per cent confluent BSR-T7/5 cells were co-transfected with eleven plasmids encoding the individual rotavirus genome segments and three helper plasmids encoding two vaccinia virus capping enzyme subunits, as well as a small membrane fusion protein using TransIT-LT1 transfection reagent (Mirus Bio, Madison, WI, USA). After 24 hours, the transfected cells were washed twice with PBS, and fresh medium without serum was added. Forty-eight hours later, MA-104 cells and trypsin (PAN-Biotech, Aidenbach, Germany; 2 µg/ml final concentration) were added. After 3 days, the co-cultured cells were frozen and thawed once before passaging of clarified supernatants (freeze-thaw supernatants) on MA-104 cells. For passaging, trypsin was added to the entire freeze-thaw supernatants (10 µg/ml final concentration), and the mixture was incubated for 1 hour at 37°C. Confluent MA-104 cells grown in 6-well plates were washed twice with PBS and the freeze-thaw supernatants were directly added without further dilution. After 1 hour, the mixture was removed, the cells were washed once, and fresh media (without serum) supplemented with trypsin (PAN-Biotech, 1 µg/mL final concentration) were added. The cells were incubated for 7 days before they were passaged again following the same protocol. At the end of the second passage, viral RNA was extracted from freeze-thaw supernatants with the NUCLISENS easyMAG system (bioMérieux, Marcy-l’Étoile, France) and digested with RNase-free DNase (Roche, Basel, Switzerland) according to the manufacturer’s instructions. RVA-RNA was detected by RT-qPCR as described previously (Falkenhagen et al. 2019).

## 3. Results

### 3.1 RVA-RNA detection in common shrew samples

A total of sixteen intestinal samples of common shrews from five trapping areas within the federal state Baden-Wuerttemberg in Germany (details in Supplementary Data S1) were analysed for the presence of RVA-RNA. Applying two widely used real-time RT-PCR protocols for the detection of mammalian RVA strains, all samples tested negative. Therefore, the more broadly reacting primers Shrew RVA-s and Shrew RVA-as, with proposed binding sites on several mammalian, avian, and an already known common shrew RVA strain, were designed (Supplementary Data S2). Using a conventional RT-PCR with these primers, two of the samples resulted in an RT-PCR product of the expected length. Sample KS14/269 originated from a female shrew and sample KS13/718 from a male shrew, both trapped at different areas. The body weights of the infected animals were 7 g and 6 g, which are among the lowest weights of the analysed animals.

### 3.2 Genome sequencing of RVA strains KS14/269 and KS13/718

Samples KS14/269 and KS13/718 were subjected to NGS, followed by *de novo* assembly of contigs and identification of rotavirus-specific sequences. Approximately 96 per cent and 83 per cent of the genomes could be sequenced with mean sequence coverages of 155–1,077 and 28–157 for the contigs of samples KS14/269 and KS13/718, respectively. Missing sequences were mainly located at the genome segment termini. For further completion of the genome sequence, sample KS14/269 was selected and all missing sequences of the ORFs were amplified using conventional RT-PCRs followed by Sanger sequencing. In addition, missing non-coding sequences of the termini of Genome Segments 4, 6 and 7, which were intended to be used in reverse genetics system, were determined using RT-PCR amplification and application of a RACE strategy. The resulting sequences of the non-coding segment termini are presented in Table 1, also showing that for seven of the twenty-two ends, the respective sequences could not be determined. From the available data, typical conserved sequence motifs common for RVA strains can be identified, including the sequence GGC(A/U)_n_ at the 5ʹ-terminus (according to the positive-sense RNA strand) and the sequence UGUGACC at the 3ʹ-terminus of the genome segments.

**Table 1.** Non-coding region nucleotide sequences at the genome segment termini of RVA strain KS14/269. The positive-sense RNA strand sequences are shown. The completely conserved nucleotides of the segment termini are shown in bold face. Start and stop codons of the open reading frames are underlined.

Genome segment (gene product)	5ʹ-terminus	3ʹ-terminus
Segment 1 (VP1)	**GGC**UAUUUACGAUG	UAAAGCUCACUUAA**UGUGACC**
Segment 2 (VP2)	n.d.	UAAGUCCUACCCACUGUGGUGA**UGUGACC**
Segment 3 (VP3)	n.d.	UAACGCUGUUUUCAGCUUAACGAGCUAACGUAAGA**UGUGACC**
Segment 4 (VP4)	**GGC**UAAAAAAUG	UAGAGCUGUUAGGAGA**UGUGACC**
Segment 5 (NSP1)	**GGC**UUUUAAAGCUCAACCAGUGGACUAACAGUGAUG	UAGUCCACUGGGCUAUGCCUGGUAGUGCGGGUUAGAAGCUUU**UGUGACC**
Segment 6 (VP6)	**GGC**UUUAAAACGAAGUCUCUGAAUUGAACAGUAUG	UGAUGUUUCUAUGUCUUGGAAGUGACUGAGAGGA**UGUGACC**
Segment 7 (NSP3)	**GGC**AAUCAAUUCCUUCUUAAAAAUG	n.d.
Segment 8 (NSP2)	**GGC**UUUUAAAGCGUCUUGGUCGCGGUUUGAGCUUUGCCGCAGCGCUAUG	n.d.
Segment 9 (VP7)	**GGC**AAACUUUUAAGUAUAGCAGUAAAUCUGCUAUG	UAGAGAUCAGUCAGCAUUU**UGUGACC**
Segment 10 (NSP4)	n.d.	UAAUAAGAACGUAGAUUCUUUAUUCUUGAGGUCACUAUCGAUGAGAACUUCAAUGCUUGUUUUCCGCGAUCUGUGUCUAUCUUCGGAAGCGCAGGCCGGAUUAACCGCUGAGACCGUCGGACUUGUGCGUACAGAGUUGAAGCCCUCUGUACGUAAUCGCGUGUGGGACAGGAUCCCUUAAUCCCUAGUACCCCAACCCACUAGGUGGGCGGAUCUGGGAAUCUGAGACGUUA**UGUGACC**
Segment 11 (NSP5)	n.d.	n.d.

n.d.—not determined.

### 3.3 Genetic variability between RVA strains from common shrews

The sequence variability of RVA strains from common shrews was assessed by comparing the RVA sequence from sample KS14/269 to the partial genome sequences of sample KS13/679 as well as to available partial sequences from samples KS11/2281 and KS12/0644 (Table 2). The latter two samples also originate from common shrews trapped in Southern Germany (Johne et al. 2019). The resulting nucleotide sequence identities varied between 66 per cent and 97 per cent, dependent on the genome segment and compared strains. The results indicate that more than one genotype exists for Genome Segments 1 and 11 among the common shrew RVAs, based on available complete ORF sequences by applying the defined sequence identity cut-offs for genotyping (Matthijnssens et al. 2008b). In addition, based on a partial sequence, an additional genotype may exist for Segment 4; however, this has to be confirmed after sequencing the complete ORF of this strain. In contrast to the high nucleotide sequence variability, the deduced amino acid sequences showed markedly higher percentages of identity. Especially, that of VP6 was highly conserved with 100 per cent identity in the three analysed common shrew RVAs.

**Table 2.** Sequence variability between different RVA strains derived from common shrews in Germany. Available corresponding sequences of RVA strains from samples KS14/269, KS13/679 and KS11/2281 (KS12/0644 for Segments 9 and 10 instead of KS11/2281) were compared to each other.

Genome segment (gene product)	Aligned sequence fragment length in nucleotides (amino acids)	Sequence identity in % for nucleotides (amino acids)
Segment 1	3,065	73–79
(VP1)	(1,021)	(81–96)
Segment 2	363	85–89
(VP2)	(120)	(100)
Segment 3	729	82–94
(VP3)	(242)	(91–95)
Segment 4	417	66–94
(VP4)	(138)	(70–100)
Segment 5	471	82–94
(NSP1)	(156)	(92–96)
Segment 6	1,032	88–97
(VP6)	(344)	(100)
Segment 7	714	86–89
(NSP3)	(237)	(95–96)
Segment 8	413	85–88
(NSP2)	(137)	(95–96)
Segment 9	262	89–93
(VP7)	(87)	(90–94)
Segment 10	386	89–93
(NSP4)	(128)	(91–94)
Segment 11	472	85–91
(NSP5)	(157)	(89–95)

### 3.4 Comparison of the RVA strain KS14/269 genome to other RVA strains and genotyping

Generally, nucleotide sequence identities of RVA strain KS14/269 were low for all genome segments when compared to the RVA genotype reference strains, peaking at 68 per cent identity for Segments 1 and 2 (Table 3). Amino acid sequences also presented low sequence identities overall, ranging from 10 to 72 per cent (Table 3). The sequences were therefore submitted to the RCWG for assignment of new genotypes. With the exception of Segment 6, which belonged to the already defined common shrew RVA genotype I27 (Johne et al. 2019), novel genotypes were assigned to all of the genome segments. The final genome constellation of strain KS14/269 is therefore G39-P[55]-I27-R26-C22-M22-A37-N26-T26-E30-H26.

**Table 3.** Sequence variability between the RVA strain from common shrew sample KS14/269 and RVA prototype sequences of the different RVA genotypes. The prototype sequences defined by RCWG (https://rega.kuleuven.be/cev/viralmetagenomics/virus-classification/rcwg) were used, with the exclusion of other common shrew RVA sequences.

Genome segment (gene product)	Sequence identity in % for nucleotides (amino acids)
Segment 1	65–68
(VP1)	(69–72)
Segment 2	66–68
(VP2)	(68–72)
Segment 3	57–60
(VP3)	(44–47)
Segment 4	52–62
(VP4)	(13–21)
Segment 5	35–48
(NSP1)	(10–24)
Segment 6	61–67
(VP6)	(59–67)
Segment 7	49–61
(NSP3)	(32–42)
Segment 8	55–59
(NSP2)	(44–51)
Segment 9	57–64
(VP7)	(43–57)
Segment 10	39–48
(NSP4)	(16–21)
Segment 11	54–62
(NSP5)	(39–49)

### 3.5 Phylogenetic analysis of the common shrew RVA strains

In order to assess the phylogenetic relationship and evolution of the common shrew RVAs in the context of other RVA strains, phylogenetic trees were reconstructed for the common shrew RVA strain KS14/269 together with all RVA genotype reference strains. Sequences from the other rotavirus species RVC, RVD, and RVF were used as outgroups. In most of these phylogenetic reconstructions, the common shrew RVA branch was placed ancestral to all other RVAs (Fig. 1A, B, and Supplementary Data S3). This clustering is also evident for the segment encoding the antigenic VP4 (Fig. 1C). In contrast, the other main antigen (VP7)-encoding segment of the common shrew RVA formed a branch that is basal only to the other mammalian RVAs (Fig. 1D). For the VP2-encoding segment, the common shrew RVA clustered with avian sequences, while for the NSP5-encoding segment, the basal relationships among the major RVA clades were not resolved (Supplementary Data S3). For the NSP1-encoding segment, the common shrew RVA clusters basal to the other mammalian RVAs, whereas the avian RVAs cluster together with RVD (Supplementary Data S3), reflecting a well-known phenomenon for the avian RVA NSP1 sequences (Trojnar et al. 2010).

**Figure 1.**
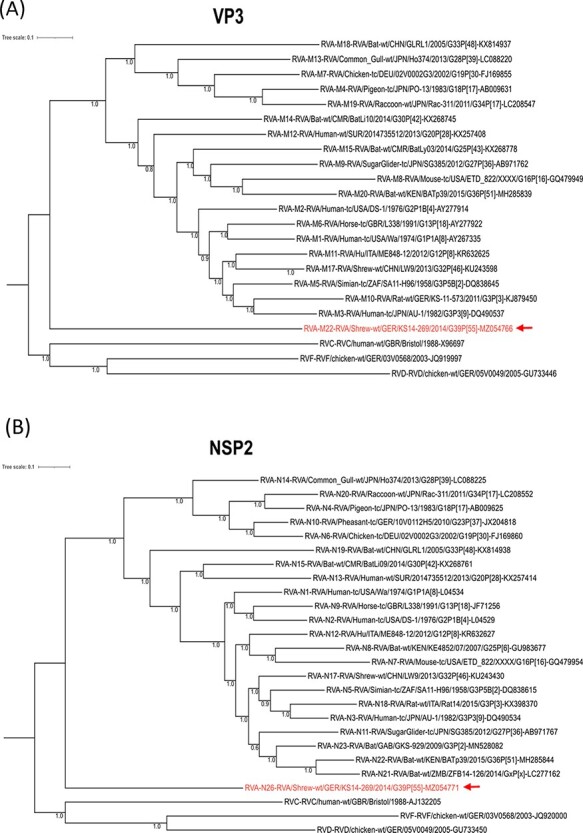
(Continued)

**Figure 1.**
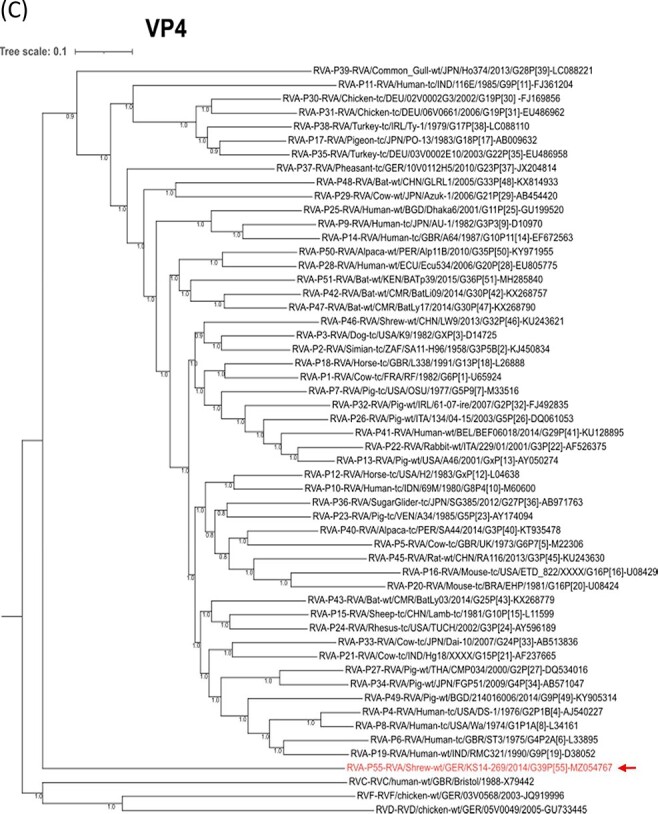
(Continued)

**Figure 1.**
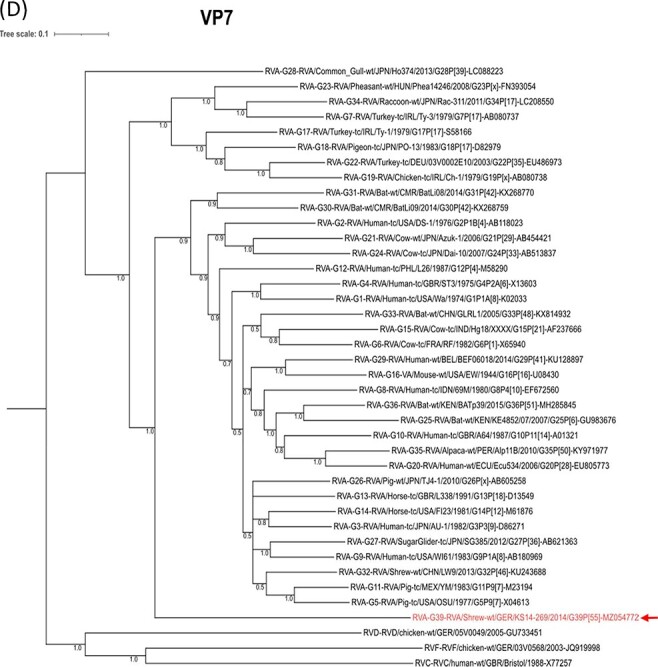
Phylogenetic relationship of the common shrew rotavirus A (RVA) strain KS14/269 with genotype reference strains based on nucleotide sequences of the complete open reading frames of genome segments encoding VP3 (A), NSP2 (B), VP4 (C), and VP7 (D). The rotavirus species, genotypes, and strain designations, including host species and GenBank accession numbers, are indicated at the branches of the tree. The genes of rotavirus C (RVC), D (RVD) and F (RVF) strains are included as outgroups. The common shrew virus strain from this study is marked in red and with an arrow. Bayesian posterior probabilities are included for all nodes. The scale bar on top shows evolutionary distance in substitutions per nucleotide.

**Figure 2.**
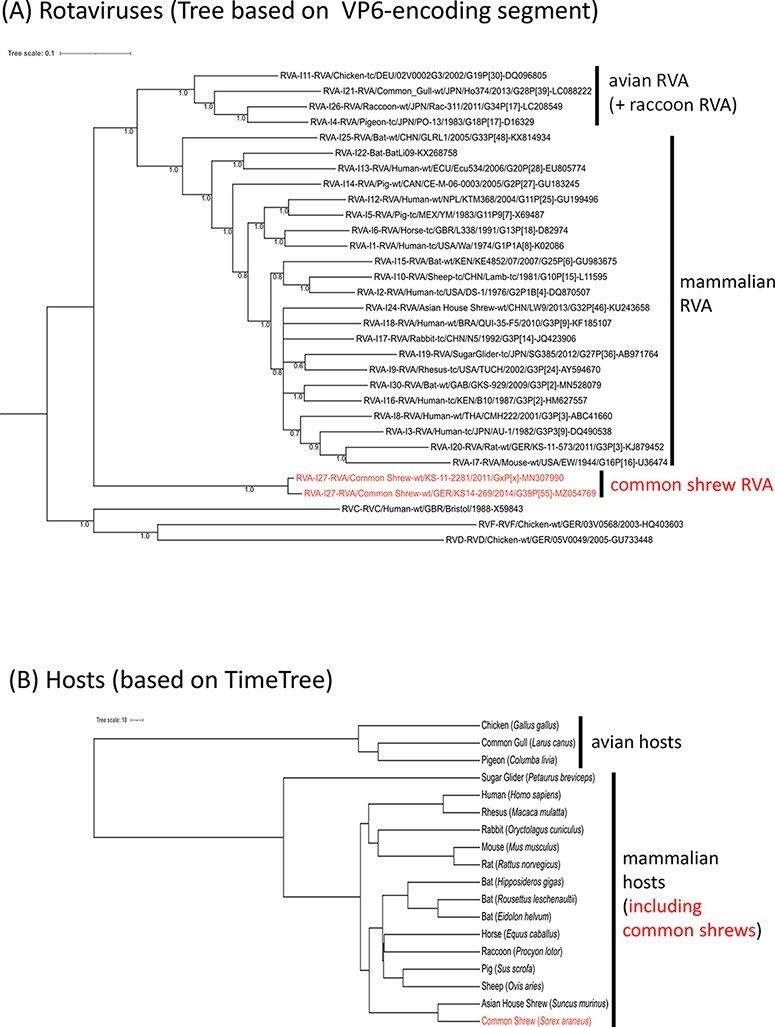
Comparison of RVA phylogeny with that of the host species. (A) Phylogenetic relationship of the common shrew rotavirus A (RVA) strains KS14/269 and KS/11/2081 with genotype reference strains based on nucleotide sequences of the complete open reading frame of the genome segment encoding VP6. Labeling of the branches of the tree is according to Fig. 1. The host species are indicated within the strain designations at the branches of the tree. The scale bar on top shows evolutionary distance in substitutions per nucleotide. (B) Phylogenetic relationship of the RVA host species based on TimeTree. The branches are labeled with the species designations according to Fig. 1 and with the scientific species names, for example, *Sorex araneus* for the common shrew. The scale bar of the species tree shows the estimated time in million years ago (mya) since the most recent common ancestor.

In order to compare the phylogeny of RVAs with that of their host species, a tree based on the VP6-encoding segment (representing a highly conserved segment also used for classification into rotavirus species; Matthijnssens et al. 2012) of the RVA genotype reference strains and a tree showing the relationships among hosts of these reference strains were generated. For the RVAs, a clade of avian RVAs is evident, also containing a raccoon-derived RVA. Other RVAs, including one strain from Asian house shrews, clustered into a main branch containing mammalian RVAs. This mammalian RVA cluster and the avian RVA cluster were clearly separated from that of the two common shrew RVAs (Fig. 2A). In contrast, the topology of the host tree is different. It shows an exclusive clustering into avian and mammalian animal species, where the common shrews cluster closely together with Asian house shrews within the mammalian branch (Fig. 2B).

### 3.6 Attempts to generate reassortants of common shrew and simian RVA strains using a reverse genetics system

**Figure 3.**
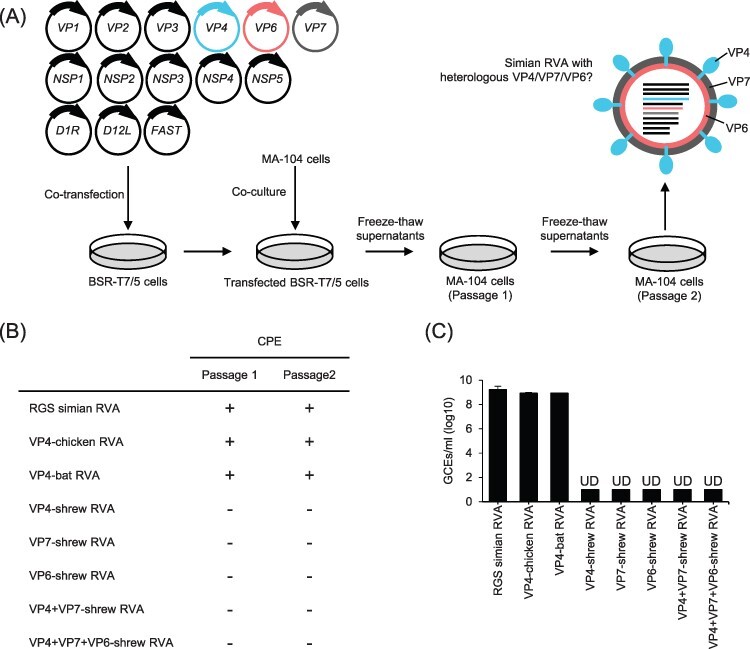
Attempts to generate reassortants using a plasmid-based reverse genetics system. (A) Schematic representation of the applied method. T7 RNA polymerase-expressing BSR-T7/5 cells are co-transfected with eleven plasmids encoding the individual RVA genome segments of simian RVA strain SA11 and three helper plasmids encoding two vaccinia virus capping enzyme subunits (D1R and D12L) as well as a small membrane fusion protein (FAST). For the generation of reassortants, the plasmids encoding VP4, VP7, and/or VP6 of simian RVA strain SA11 are substituted with corresponding plasmids of other RVA strains (shown in red, blue, and grey). MA-104 cells are added directly to the transfected cells three days post-transfection, and the cells are co-cultured for an additional 3 days. After a freezing and thawing cycle, supernatants are used to infect MA-104 cells. Seven days later, virus is passaged one more time on MA-104 cells before freeze-thaw supernatants are analysed for the presence of reassortants (diagrammatically shown as a coloured particle). (B) Occurrence of CPEs after two passages in MA-104 cells. (C) Detection of viral RNA after two passages in MA-104 cells using RT-qPCR. Data are means ± standard deviation. All rescue attempts and analyses were performed twice and in duplicates; RGS simian RVA—simian RVA strain SA11 generated using the plasmid-based reverse genetics system; VP4-chicken RVA—reassortant carrying VP4 from chicken RVA strain 02V0002G3 in the backbone of simian RVA strain SA11; VP4-bat RVA—reassortant carrying VP4 from bat RVA strain BatLy03 in the backbone of simian RVA strain SA11; VP4/VP7/VP6-shrew RVA—reassortants carrying VP4, VP7, and/or VP6 from shrew RVA strain KS14/269 in the backbone of simian RVA strain SA11; GCEs—genome copy equivalents, UD—undetected.

To analyse whether the common shrew RVA can exchange genetic material with other RVAs by reassortment, an available reverse genetics system (Kanai et al. 2017) based on the simian RVA strain SA11 was used (Fig. 3A). In this system, eleven plasmids encoding the single rotavirus genome segments under control of the T7 RNA polymerase promoter and three helper plasmids encoding capping enzymes, as well as a small membrane fusion protein were co-transfected into T7 RNA polymerase-expressing BSR-T7/5 cells. The transfected cells were co-cultured with MA-104 cells, which are highly susceptible to several RVA strains. After two additional passages in MA-104 cells, successful rescue of infectious virus was determined by monitoring the infected cells for the occurrence of a rotavirus-typical cytopathic effect (CPE) and by RVA-specific RT-qPCR.

Rescue attempts were focused on reassortants with the major antigens VP4-, VP7-, and VP6-encoding segments from shrew RVA strain KS14/269 in the backbone of simian RVA SA11. The generation of mono-reassortants for VP4, VP7, or VP6, a double VP4/VP7 reassortant, and a triple VP4/VP7/VP6 reassortant was attempted. However, neither a CPE nor a rotavirus RNA was detected at the end of the second passage in each case (Fig. 3B and C), indicating that no viable reassortants could be rescued. In contrast, both a CPE and viral RNA could readily be detected when rescue of simian RVA strain SA11 using the system was attempted (Fig. 3B and C). In another control experiment, we were able to rescue viable reassortants carrying VP4 from chicken RVA strain 02G0002G3 or VP4 from bat RVA strain BatLy03 in the backbone of the simian RVA strain SA11 using the system (Fig. 3B and C).

## 4. Discussion

RVA strains are known to exhibit a high degree of genetic diversity. In addition to point mutations and gene rearrangements, the process of reassortment enables a rapid change in RVA genome constellations, which also may involve the major antigen-encoding genome segments (Kirkwood 2010). As zoonotic transmission of RVAs is possible, animal RVAs could play an important role for human RVA epidemiology (Martella et al. 2010). In contrast to domestic animals, wild animals have been scarcely analysed for the presence of RVAs thus far (Matthijnssens et al. 2009; Badaracco et al. 2013; Sachsenröder et al. 2014; Moutelíková et al. 2016; Niendorf et al. 2021). Recently, several RVAs have been identified in wild bats, which showed close genetic relationships to rare human RVA strains, suggesting bats as the origin of these strains (Simsek et al. 2021). In our study, RVAs from wild-living common shrews, which are known reservoirs for other zoonotic pathogens (Schlegel et al. 2012; Fischer et al. 2018a), were genetically and biologically characterized in detail to determine their relationship to other RVAs and to assess their zoonotic potential.

The sequencing of the nearly complete genome of an RVA strain from common shrews enabled us to compare it with a wide range of available other RVA genome sequences. Typical features, such as RVA consensus sequences at the non-coding 5ʹ- and 3ʹ-ends, are present in the virus genome, and the amino acid sequence identities of the encoded VP6 with other RVA strains (>59 per cent) are higher than the defined threshold of 53 per cent (Matthijnssens et al. 2012). According to these criteria, the common shrew RVA has to be classified into species A of the *Rotavirus* genus. However, very low nucleotide sequence identities with other mammalian and avian RVAs were discovered across all segments of the viral genome, resulting in the assignment of novel genotypes for all of the genome segments. In addition, we were not able to create viable reassortants containing genome segments of the common shrew strain and an RVA strain from monkey, as discussed below in more detail. This might indicate that both viruses do not belong to the same rotavirus species since a general determinant for grouping virus isolates into the same virus species within the family *Reoviridae* is their capacity to exchange genetic information by genome segment reassortment (Matthijnssens et al. 2021). Further studies on the biological and genetic properties of the common shrew RVA strains are necessary in future to reconsider a final assessment of their taxonomical classification.

The RVA strain described in this study does not represent an isolated case limited to a single infected common shrew. In combination with data from Johne et al. (2019), several additional (partial) RVA sequences from common shrews originating from different regions in Germany and sampled at several time-points have been detected. Although these strains showed a considerable degree of nucleotide sequence diversity between 3 per cent and 34 per cent, some of the genes showed a remarkably high deduced amino acid sequence identity, for example, 100 per cent for VP6, suggesting that this specific RVA lineage had fully adapted to the common shrew and had started diversification within this host. However, this does not exclude that shrews can also be infected with other RVA genotypes more closely related to non-shrew mammalian strains as recently shown for RVAs detected in the Asian House shrew in China (Li et al. 2016).

A more detailed phylogenetic analysis of the RVA sequences from common shrews was performed, including all available RVA genotype reference sequences, which reflect the overall genetic diversity of RVAs. Across all segments of the RVA genome, the common shrew strains clustered outside the majority of other RVAs. Moreover, for seven of the eleven genome segments, the common shrew strain formed a basal branch to all other mammalian and avian RVAs. This argues for a long-term separated evolution of these RVA strains in common shrews without the exchange of genetic material with other RVAs.

It is intriguing that the branching of the RVA strains is very different to that of their hosts in case of common shrews, leading to the conclusion that a simple virus-host co-divergence cannot explain all aspects of the observed RVA diversity. The factors causing the evolution of very distinct RVAs, which cluster basal to all other mammalian and avian RVAs, in shrews are not known so far. However, basal separation from other mammalian rotaviruses has already been described for other rotavirus species in common shrews. For instance, rotavirus species C, which is present in other mammals, has not been detected so far in common shrews; however, viruses clustering basal to rotavirus C, which have been putatively classified as species K, have been identified in common shrews (Johne et al. 2019). The same situation is evident for the putative rotavirus species L, which is a basally clustering relative of the mammalian rotavirus H and which has only been detected in common shrews so far (Johne et al. 2019). Further studies are necessary to elucidate the causes of the evolution of highly divergent rotavirus strains in common shrews, which may include the analysis of specific molecular host factors or special features in behaviour or habitat structure for this animal species.

Despite the low genome sequence identity to other RVA strains, the common shrew RVA contained the main functional genetic RVA motifs; therefore, a reassortment with other RVAs could be assumed. In our study, we analysed the reassortment potential using an established reverse genetics system, which previously had been successfully used for the generation of reassortants with exchanged VP4- and VP7-encoding genome segments from different human, other mammalian, and avian strains (Falkenhagen et al. 2019, 2020; Patzina-Mehling et al. 2020). However, no viable reassortants could be generated using the common shrew genome segments encoding the major antigens VP4, VP7, or VP6 in the backbone of the simian RVA strain SA11. This might indicate a low reassortant potential of the common shrew strain with other mammalian strains. However, the applied system is only based on the SA11 strain, and recently developed reverse genetics systems for other RVA strains (Uprety, Wang, and Li 2021) should therefore be tested in future. In addition, it has been shown that the applied system is restricted to the presence of a specific receptor-binding region in the analysed VP4 molecules (Falkenhagen et al. 2021), which may be absent in the common shrew strain. For the simian RVA strain RRV, the VP4 amino acid residues that play a role in receptor binding are known (Dormitzer et al. 2002). In comparison to VP4 of the simian RVA strain RRV and VP4 from the other rescuable VP4 reassortants, the amino acid sequence of the shrew RVA strain differed in five out of eight amino acid residues that are important for receptor binding (not shown). However, other reasons, such as incompatibilities at the VP4-VP7-VP6-VP2 interfaces (Heiman et al. 2008; McDonald et al. 2009) or different secondary RNA structures (McDonald and Patton 2011), may have negatively affected reassortment. In future, the reasons for the failed reassortment could be examined by the generation of chimeric genome segments.

In conclusion, we detected and characterized highly divergent RVA strains from common shrews, which further increase our knowledge about the high genetic variability of RVAs. Phylogenetic analysis suggests that the evolution of these viruses differs from those of other mammalian and avian RVAs. Our experiments based on a reverse genetics system suggest a low potential for the reassortment of the common shrew strains with other mammalian RVAs. Taken together, the results suggest a rather low zoonotic potential of the common shrew RVAs; however, additional studies on the transmissibility to other animals and humans may be necessary. Generally, wild animals should be increasingly considered as sources for rotavirus infections, and future research should focus on the assessment of RVA diversity in wild animals and characterization of the zoonotic potential of identified strains.

## Supplementary Material

veac004_Supp

## Data Availability

The GenBank accession numbers of the nucleotide sequences of the shrew rotaviruses identified in this study are presented in Section 2.3.
