# Supplementary material for: Genetic and biological characteristics of species A rotaviruses detected in common shrews suggest a distinct evolutionary trajectory
Source: Virus Evol. 2022 Jan 28;8(1):veac004. doi: 10.1093/ve/veac004 (PMC8838746; doi:10.1093/ve/veac004)

**Supplementary Data**

**Genetic and biological characteristics of species A rotaviruses from common shrews suggest a distinct evolutionary trajectory**

**Alexander Falkenhagen, Simon H. Tausch, Anton Labutin, Josephine Grützke, Gerald Heckel, Rainer G. Ulrich, Reimar Johne**

**Supplementary Data S1:** Analysed samples of common shrews (*Sorex araneus*) trapped in areas A – E of Baden-Wuerttemberg region of Germany. The rotavirus A-positive animals are indicated in bold face.

| Sample designation | Sex | Weight (g) | Trapping area | Trapping time point (month/year) |
| --- | --- | --- | --- | --- |
| KS13/613 | Male | 6 | A | 04/2012 |
| KS13/614 | Male | 7 | B | 04/2012 |
| **KS13/679** | **Male** | **6** | **C** | **09/2012** |
| KS13/718 | Female | 8 | B | 10/2012 |
| KS14/230 | Female | 11 | D | 06/2013 |
| KS14/233 | Female | 8 | A | 10/2013 |
| KS14/234 | Male | 6 | C | 032013 |
| KS14/235 | Male | 7 | C | 03/2013 |
| KS14/236 | Male | 11 | C | 08/2013 |
| KS14/237 | Male | 7 | E | 08/2013 |
| KS14/247 | Male | 8 | B | 11/2013 |
| KS14/261 | Male | 7 | C | 08/2013 |
| KS14/266 | Male | 7 | A | 10/2013 |
| KS14/267 | Male | 7 | A | 10/2013 |
| **KS14/269** | **Female** | **7** | **A** | **08/2013** |
| KS14/307 | Female | 9 | D | 08/2013 |

**Supplementary Data S2:** Primers used in the study.

| **Designation** | **Sequence (5’-3’)** | **Application** |
| --- | --- | --- |
| Shrew RVA-s | CGATGTCATCAGCGTCAAATGG | RVA detection in shrews  (387 bp VP1 gene amplicon) |
| Shrew RVA-as | CCCATTGCGACACGTCAGTGTA |  |
| S4-173-as | CTTCCGCTGCCCCATTCAAC | RACE for 5’-terminus of segment 4 |
| S4-200-as | TATTCAACAGGTATTTGTTCAC |  |
| S2-1-17-s | TGTCAGGCTATTAAAGGCTCAA | 5’-end determination of VP2-ORF |
| S2-200-as | CTTTGTCACTACGTTCTTTCTC |  |
| S3-1-12-s | ACGTGTCAGGCTATTAAAGC | 5’-end determination of VP3-ORF |
| S3-210-as | ATGTACAGCACGTTATGAGTCG |  |
| S10-20-40-s | CCGAGAGAGCGCGTGCGGAAAG | 5’-end determination of NSP4-ORF |
| S10-210-as | CCACTGGCGACTGTCTTTGCAC |  |
| S6-1-12-s | ACGTGTCAGGCTTTAAAACG | Confirmation of 5’-terminal sequence of segment 6 |
| S6-200-as | AATGGGCAATAAGCCAATCC |  |
| S9-1-12-s | ACGTGTCAGGCAAACTTTTA | Confirmation of 5’-terminal sequence of segment 9 |
| S9-200-as | CAAAATTTTGAGCGTTAGCAG |  |

**Supplementary Data S3.** Phylogenetic relationship of the VP1-, VP2-, NSP1-, NSP3-, NSP4- and NSP5-encoding genome segment sequences of common shrew RVA strain KS14/269 with the corresponding sequences of all respective RVA genotype reference strains. The rotavirus species (RVA-RVF), genotypes, strain designations and GenBank accession numbers are indicated at the branches of the trees. The shrew virus strain from this study is marked in red. Bayesian posterior probabilities are included for all nodes. The scale bar on top shows evolutionary distance in substitutions per nucleotide.


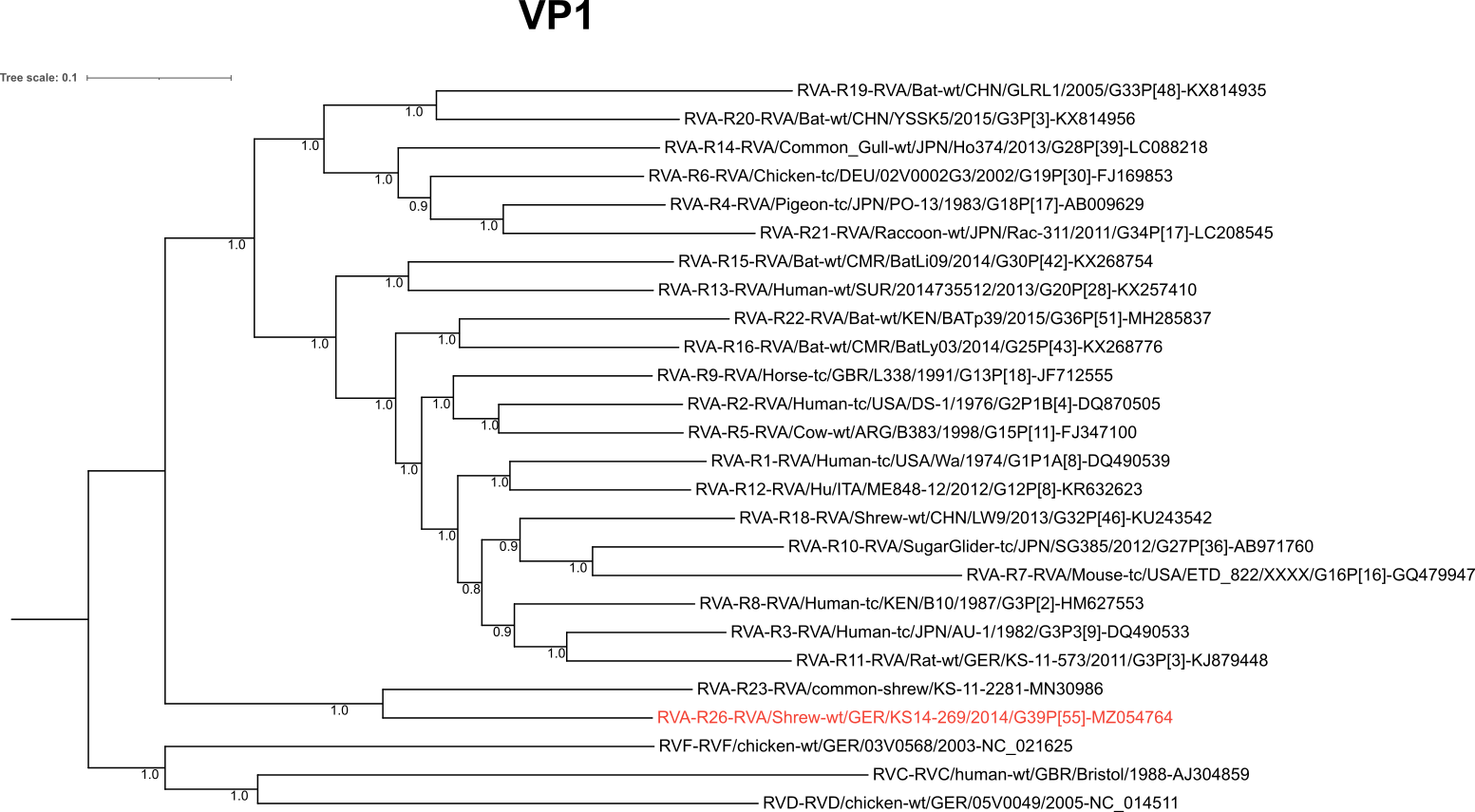


**
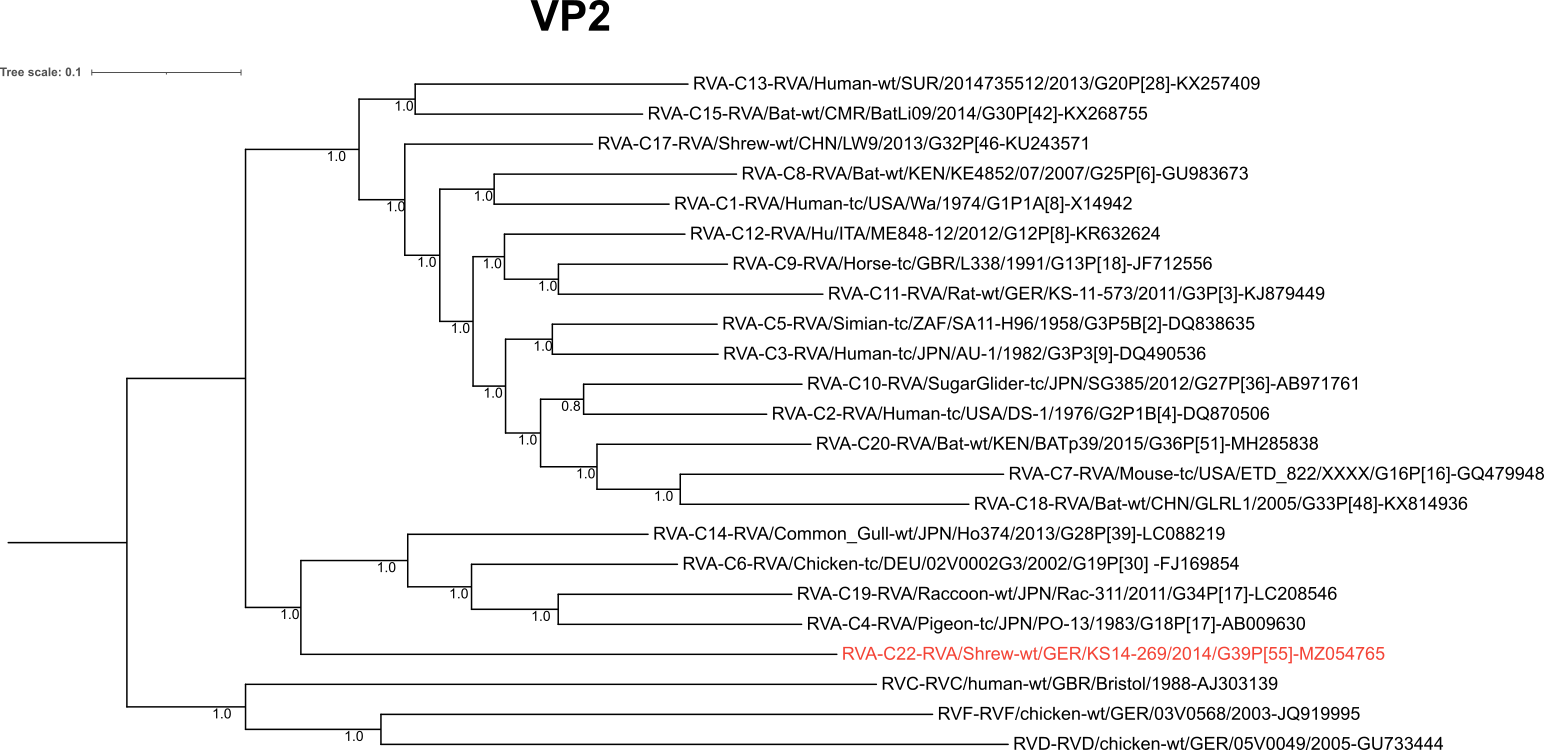
**

**Supplementary Data S3 (cont.)**
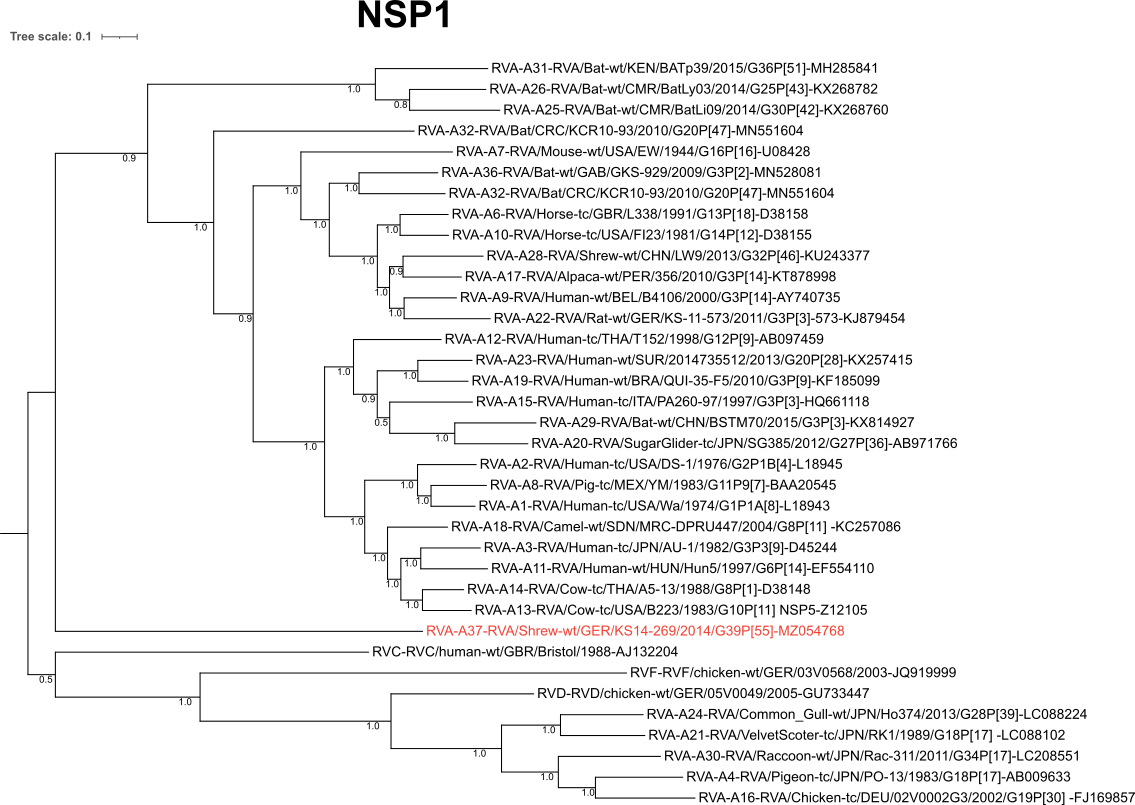


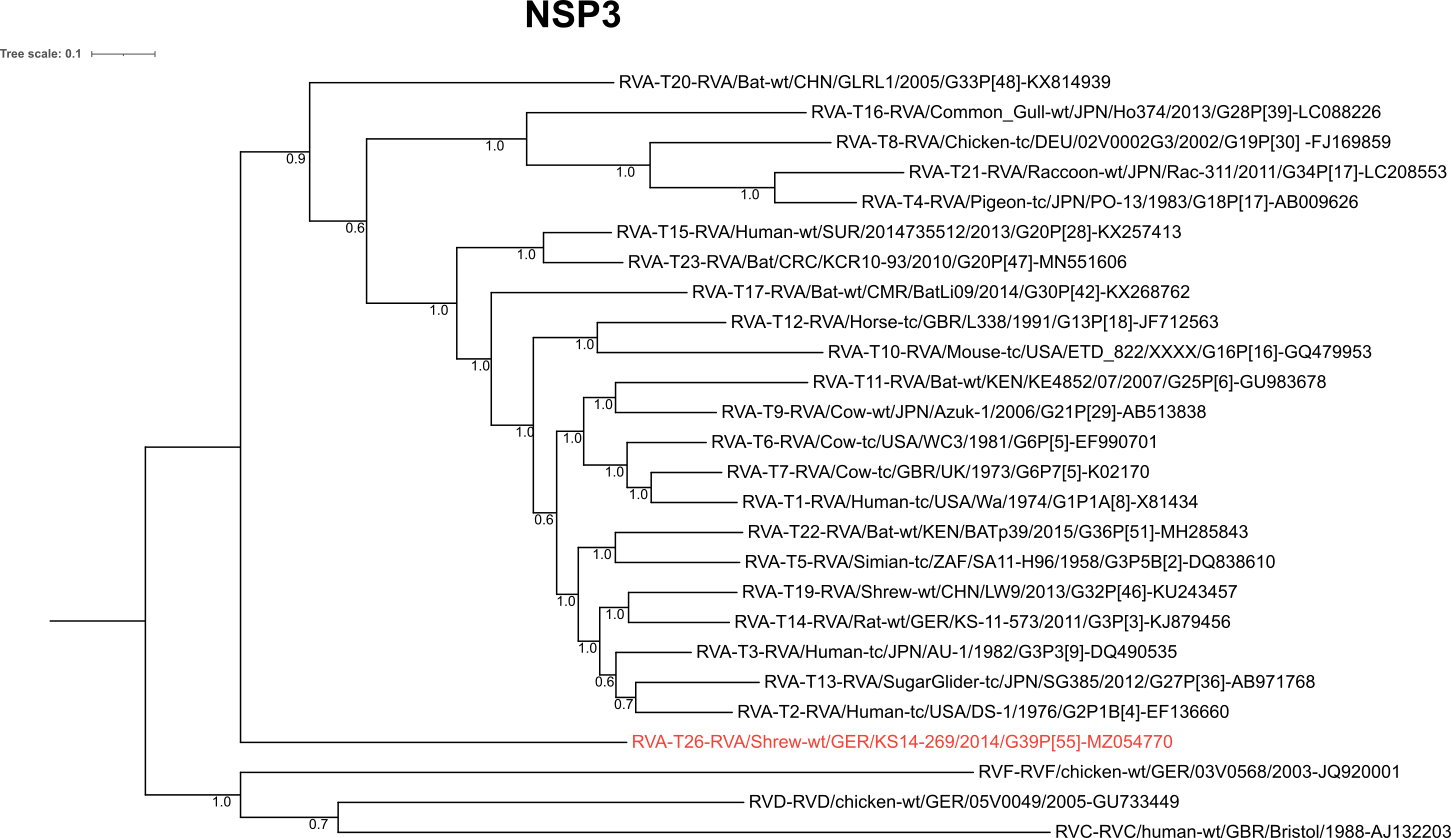


**Supplementary Data S3 (cont.)**

**
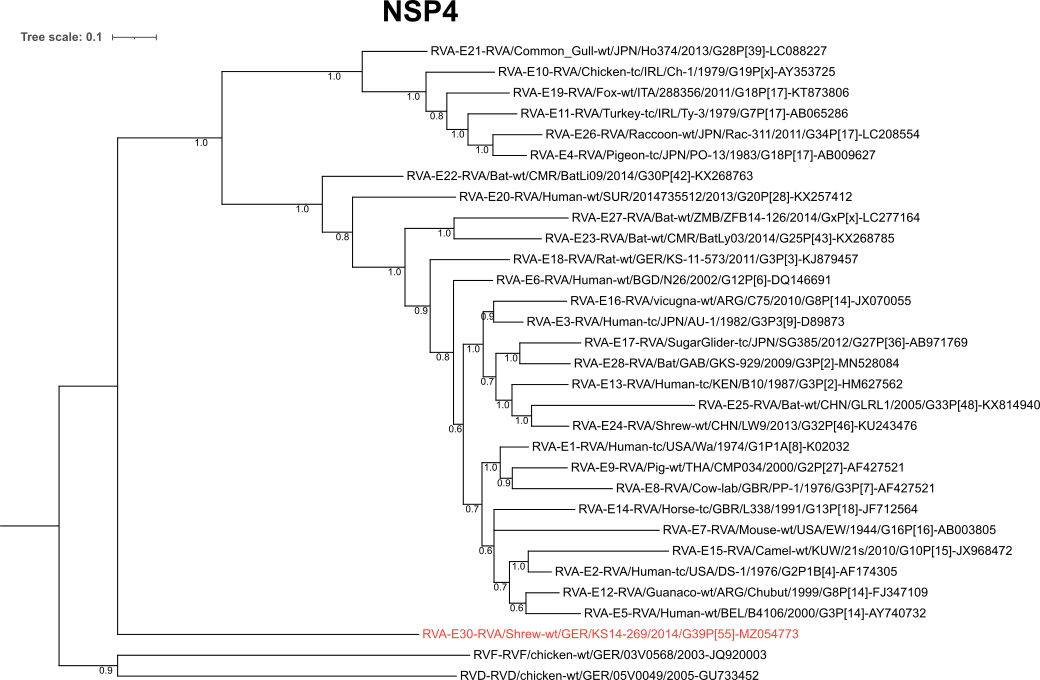
**


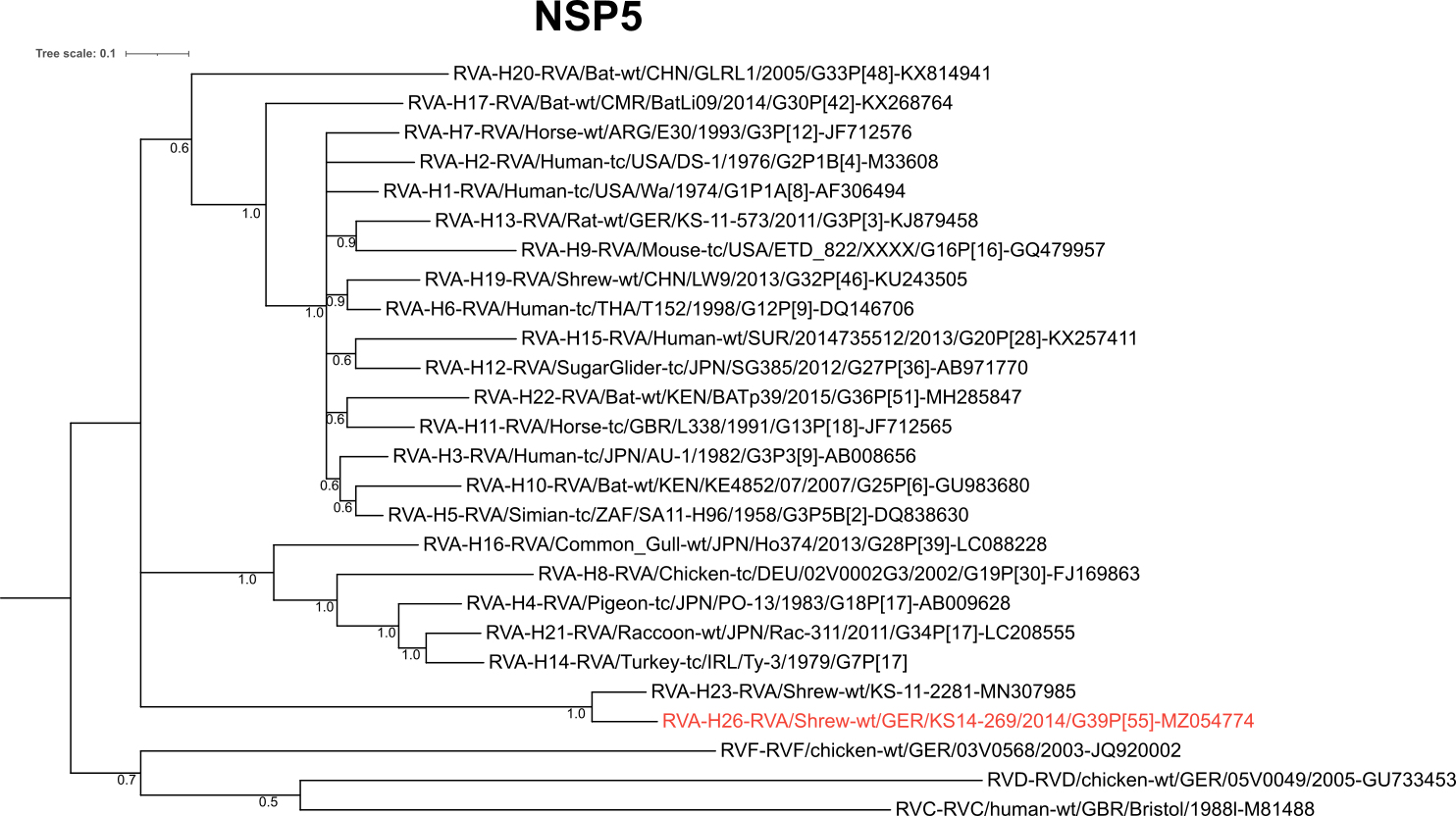

Supplement: veac004_Supp [file veac004_supp.zip › revised2 Supplementary data.docx]
